# What Makes Mental Modeling Difficult? Normative Data for the Multidimensional Relational Reasoning Task

**DOI:** 10.3389/fpsyg.2021.668256

**Published:** 2021-05-06

**Authors:** Robert A. Cortes, Adam B. Weinberger, Griffin A. Colaizzi, Grace F. Porter, Emily L. Dyke, Holly O. Keaton, Dakota L. Walker, Adam E. Green

**Affiliations:** ^1^Department of Psychology, Georgetown University, Washington, DC, United States; ^2^Center for Neuroaesthetics, University of Pennsylvania, Philadelphia, PA, United States; ^3^Interdisciplinary Program in Neuroscience, Georgetown University, Washington, DC, United States

**Keywords:** relational reasoning, mental model, difficulty, multidimensional, normative, mixed-effects

## Abstract

Relational reasoning is a complex form of human cognition involving the evaluation of relations between mental representations of information. Prior studies have modified stimulus properties of relational reasoning problems and examined differences in difficulty between different problem types. While subsets of these stimulus properties have been addressed in separate studies, there has not been a comprehensive study, to our knowledge, which investigates all of these properties in the same set of stimuli. This investigative gap has resulted in different findings across studies which vary in task design, making it challenging to determine what stimulus properties make relational reasoning—and the putative formation of mental models underlying reasoning—difficult. In this article, we present the Multidimensional Relational Reasoning Task (MRRT), a task which systematically varied an array of stimulus properties within a single set of relational reasoning problems. Using a mixed-effects framework, we demonstrate that reasoning problems containing a greater number of the premises as well as multidimensional relations led to greater task difficulty. The MRRT has been made publicly available for use in future research, along with normative data regarding the relative difficulty of each problem.

## Introduction

Relational reasoning is a complex form of human cognition involving evaluation of relations between representations (Goodwin and Johnson-Laird, 2005; Knauff, 2009). Relational reasoning is closely linked to fluid intelligence (Crone et al., 2009) and problem solving in novel situations (Cattell, 1971; Halford et al., 1998). Other work indicates relational reasoning as a contributor to social development (Holyoak and Thagard, 1997; Green et al., 2017), learning (Gentner, 1983; Knowlton et al., 2012), and creativity (Green et al., 2010; Green, 2016, 2018; Weinberger et al., 2016).

One of the more popular lab-based assessments of relational reasoning involves presenting participants with verbal reasoning problems consisting of a set of (most commonly) two premises and a conclusion statement. Participants are tasked with evaluating whether the conclusion logically follows from the information in the premises (e.g., Premise 1: The newer liquid is denser than water/Premise 2: Water is denser than the older liquid/Conclusion: The new liquid is denser than the older one*;* de Soto et al., 1965; Johnson-Laird, 1972).

While a number of neurocognitive mechanisms for relational reasoning have been offered, one of the most prominent accounts is Mental Model Theory (MMT; Johnson-Laird, 2001, 2010). According to MMT, humans are able to manipulate and represent information for reasoning and problem solving (Johnson-Laird, 2001, 2010) by leveraging the brain's evolved visuospatial resources. Thus, when solving relational reasoning problems, reasoners build “mental models” of the essential pieces of information conveyed in the premises, with their relations to each other represented spatially (Roberts, 2000; Johnson-Laird, 2001, 2010). Further, visuospatial representations—i.e., “mental models”—are utilized even when the information contained within a relational reasoning problem is not intrinsically spatial. For instance, in the sample problem provided above, a reasoner may internally represent a “the newer liquid” as spatially above “water,” even though the concept of “density” is not inherently spatial.

Crucially, taking a spatially-based approach, such as building mental models, supports higher accuracy on relational reasoning problems (Galotti et al., 1986; Roberts, 2000; Robinson and Hertzog, 2003; Schaeken et al., 2014). This evidence accords with prior work indicating that experts within a given domain are able to generate mental models with higher representational accuracy and complexity compared to novice representations (Gadgil et al., 2012).

An important topic of inquiry is identifying which factors can influence the extent to which people are able to form and apply mental models while reasoning. Prior work has indicated a number of ways to make relational reasoning problems more difficult. For example, the inclusion of additional premises has been shown to increase problem difficulty because additional premises increase the demand on working memory by necessitating the construction of a more complex mental model (Klauer, 1997; Johnson-Laird, 2001). Indeed, Goodwin and Johnson-Laird (2005) found that three premise relational reasoning problems yielded more accurate responses than four premise problems with the same conclusions.

Another factor that influences problem difficulty—and, presumably, the ease with which individuals are able to construct and apply mental models—is the number of dimensions specified in the relations. Multiple studies have indicated that relational reasoning problems with one dimension of relations (e.g., “Bob is to the left of Joe”) are easier to solve than problems with two dimensions of relations (e.g., “Bob is above and to the left of Joe”; Johnson-Laird, 1972, 1989). In addition, relational reasoning problems with indeterminate solutions (i.e., the conclusion cannot be logically determined because the relation between objects is indeterminate, as in the following problem: Premise 1: Bob is to the left of Joe/Premise 2: Rick is to the left of Joe/Conclusion: Bob is to the left of Rick) are more difficult to solve than problems with determinately true or false conclusions (Byrne and Johnson-Laird, 1989; Schaeken et al., 2007). Indeterminate problems are likely to be more challenging because they necessitate the construction of multiple models (e.g., multiple possible constructions of the indeterminate relation) to reach the correct answer (Byrne and Johnson-Laird, 1989; Schaeken et al., 2007).

Furthermore, the order of the premises within a relational problem may also influence mental model construction and task difficulty. Prior work has demonstrated that the location of crucial premises (i.e., premises that provide information necessary to determine the validity of the conclusion) can impact problem difficulty, as displaying the crucial premise first requires the construction of only one model, whereas displaying an irrelevant premise (i.e., has information not needed to solve the conclusion) first requires the construction of multiple models (Henst, 1999). In contrast, however, other studies have found that problems with continuous premises (Premise 1: Bob is to the left of Joe/Premise 2: Joe is to the left of Rick) are not more challenging than problems with discontinuous premises (Premise 1: Bob is to the left of Joe/Premise 2: Rick is to the right of Joe) (Vandierendonck, 1996; Henst, 1999). The language used to describe relations between objects may also impact problem difficulty. Knauff and Johnson-Laird (2002) demonstrated that stimuli that are easy to visualize but difficult to spatially envision (e.g., “the dog is *dirtier* than the cat”) can actually impede the reasoning process, while problems with relations described in non-spatial terms (e.g., “the dog is *dumber* than the cat”) are just as difficult as problems with relations described in spatial terms (e.g., “the dog is *above* the cat”; Carreiras and Santamaria, 1997; Knauff and Johnson-Laird, 2002). Lastly, research from the field of behaviorism has put forth the Relational Frame Theory (Blackledge, 2003), which claims that distinguishing relations between stimuli is a core component of symbolic cognition that in particular supports deductive relational reasoning as well as intelligence more broadly (Blackledge, 2003; Tonneau, 2004). Recent RFT-based research has explored other aspects that impact relational reasoning problem difficulty, such as equivalency (same vs. different) and time (before vs. after) (McLoughlin et al., 2020).

Collectively, these findings provide some evidence indicating how stimulus properties of relational reasoning problems may influence mental model formation and/or task difficulty. However, to our knowledge, there has not yet been a comprehensive study to investigate all of these properties within the same set of stimuli. This has resulted in different findings across studies which vary in task design, making it challenging to determine the relative—or cumulative—effect of modifying different stimulus properties. Here, we present the Multidimensional Relational Reasoning Task (MRRT), a task which systematically varied the following stimulus properties of relational reasoning problems: Number of Premises (2 or 3), Number of Dimensions (1 or 2), Relation Type (Spatial or Non-spatial), Solution (True, False, or Indeterminate), Premise Order (Continuous or Discontinuous), and Conclusion Phrasing (“A first” or “A second”). The MRRT is publicly available for use in future research, along with normative data regarding the relative difficulty of each problem(https://osf.io/qfvp2/).

## Materials and Methods

### Participants

A total of 321 participants were recruited through Prolific.ac (Palan and Schitter, 2018), and compensated $12 for their participation. Participation was limited to adults ages 18–36 living in the United States. Substantial data removal is standard in online data collection (Allahbakhsh et al., 2013; Buhrmester et al., 2015; Palan and Schitter, 2018), and was anticipated in the present study. We included four attention check items (e.g., please select “True”) throughout the study to screen for participants who were not properly attending to the questions (e.g., rushing through and clicking answers). Eight participants were removed for missing one or more attention checks, and three participants were removed because they did not complete the entire study. Therefore, the final sample included 310 participants (43% Female, 55% Male, 2% Other; mean age = 26.75 years, SD = 4.85; 63.5% Caucasian, 12.6% Asian, 7.5% African American, 6.1% Hispanic; 0.7% Native American, 7.7% Mixed Race, 1.9% Other; Total Years of Education: 40.9% 16+ years, 40.9% 13–15 years, 15.5% 12 years, 2.6% 0–11 years). All study procedures were approved by the Georgetown University Institutional Review Board, and all participants provided informed written consent before participation.

### Design

The MRRT comprised 90 total reasoning problems. Data were collected using a planned missing data design to limit the time necessary for participants to complete the study in an effort to improve data quality by reducing participant fatigue and minimizing missingness due to attrition (Graham et al., 2006; Little and Rhemtulla, 2013). We utilized the 3-form design (Graham et al., 1994, 1996, 2001), such that the 90 problems were divided into four different sets of 22–23 problems (X, A, B, C); each set had the same number of each problem type (e.g., every set had half non-spatial problems, half false problems, half two dimensions, etc.). We then randomly sorted participants into three different groups of ~100 participants each to create a Missing Completely at Random (MCAR) design (Heitjan and Basu, 1996). In line with prior research (Graham et al., 2006; Little and Rhemtulla, 2013), all groups completed the X set and then each of the three groups completed two of the A, B, or C sets; thus, each participant completed 67 total problems, with the same number of each problem type. Group 1 (*N* = 105) completed X, A, B; Group 2 (*N* = 102) completed X, A, C; Group 3 (*N* = 103) completed X, B, C. This meant that every problem was completed by at least 200 participants, with problems from the X set being completed by all 310 participants.

### Procedure

Participants completed the MRRT as part of a larger study that included additional cognitive tasks and personality surveys; the entire study lasted roughly 1.5 h. Within the testing session, MRRT was always completed first. The MRRT was administered in three separate blocks (one for each set), with mandatory 3 min breaks between each block to prevent participant fatigue. After the MRRT was completed, the remaining tasks were administered in a random order, with the demographics survey always administered at the end.

### Multidimensional Relational Reasoning Task

The Multidimensional Relational Reasoning Task (MRRT; available for use at https://osf.io/qfvp2/) consisted of 90 reasoning problems that systematically varied the following stimulus properties: Number of Premises (2 or 3), Number of Dimensions (1 or 2), Relation Type (Spatial or Non-spatial), Solution (True, False, or Indeterminate), Premise Order (Continuous or Discontinuous), and Conclusion Phrasing (“A first” or “A second”). Each problem was composed of 2 or 3 premises and a conclusion (which was shown in all capital letters), and participants were instructed to respond with “True” if the conclusion necessarily followed from the premises, or “False” if the conclusion could possibly be false (i.e., if the solution is indeterminate). Participants were instructed to solve every problem in their head without the use of pencil/paper or their fingers and were told they would complete the task in three separate blocks, each separated by mandatory 3 min breaks. Time was unlimited for each MRRT problem because stimulus properties varied widely, and normative reaction times for these problems was not previously known—therefore, one goal of this study was to collect normative reaction time data on this stimulus set to understand the time it took to solve each problem without constraint.

**Figure 1:**
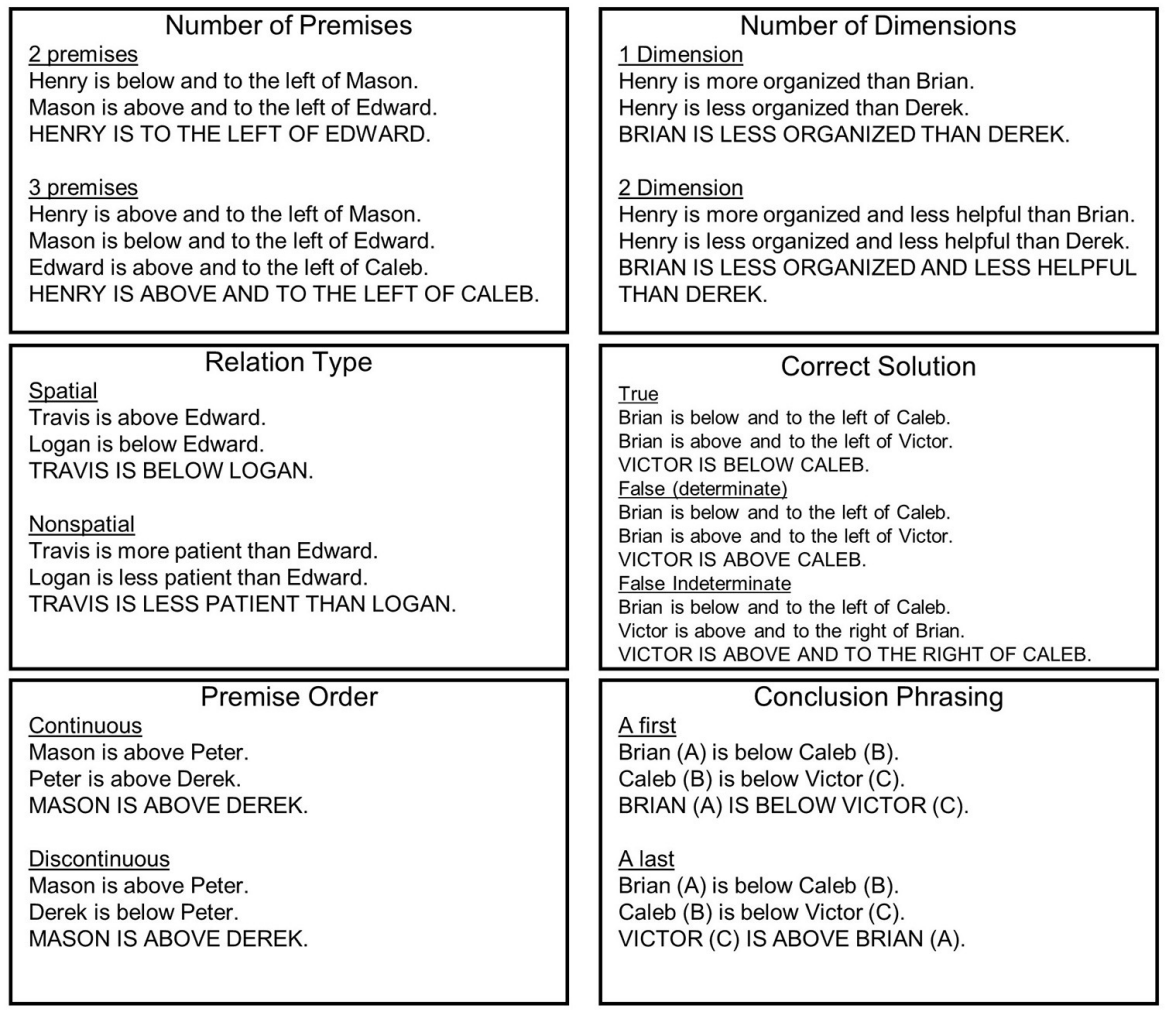
Stimulus properties systematically varied in the MRRT.

Figure 1 provides an illustration of the manner in which different stimulus properties were varied. The particular non-spatial words were equally utilized across all non-spatial problems. Half of the problems had two premises, half of the problems had three premises. One-third of the problems used only one dimension when describing relations between names (e.g., for spatial problems, saying only “above/below” or only “left/right”; for non-spatial problems, saying only “more/less certain”), and two-thirds of the problems used two dimensions when describing relations between names (e.g., for spatial problems, using “above and to the right”; for non-spatial problems, using “more certain and less excited”). Half of the problems used spatial relations (above, “below,” “to the left of,” “to the right of”) and half of the problems used non-spatial relations (“more/less excited,” “organized,” “patient,” “helpful,” “realistic,” “certain”). One-third of the problems had a conclusion which was determinately true (i.e., necessarily followed from the premises), one-third of the problems had a conclusion that was determinately false (i.e., necessarily false based on the premises) and one-third of the problems had indeterminate conclusions (i.e., could not be confirmed or denied by premises). One-fourth of the problems had a continuous premise order and three quarters of the problems had a discontinuous premise order. Lastly, the phrasing of the conclusion was varied across all problems, such that half of the problems had the “A” name come first, and half of the problems had the “A” name come last. The names used in the MRRT were 12 two-syllable male names from ranks 50–100 in the list of most popular names in the 1990s (https://www.ssa.gov). Further explanation of all these stimulus properties can be found at https://osf.io/qfvp2/.

Every problem was completed by at least 200 participants, with problems from the X set being completed by all 310 participants. See https://osf.io/qfvp2/ for the full dataset and normed data for each reasoning problem. However, an error in data collection led to missing data for four of the 90 reasoning problems. These problems were thus not included in analyses, but are still included among the publicly available stimuli without normed data.

### Analytic Strategy

In order to assess the impact of stimulus properties on task performance (i.e., RT and accuracy), we conducted a series of mixed-effects models. Mixed-effects models are appropriate when several repeated measurements or observations (Level 1) are nested within a higher level of data (Level 2; Longford, 1995; Goldstein, 2011). In the present study, stimulus properties (e.g., number of dimensions, number of premises) were modeled as a Level 1 variables, nested within each participant (Level 2). Because we were interested in examining the impact of stimulus properties on both RT and accuracy, we performed separate mixed-effects models for these two dependent variables. The effect of stimulus properties on accuracy was investigated using a mixed-effects logistic regression because accuracy was a binary variable (i.e., each individual response was either correct or incorrect). RT models were estimated via mixed-effects linear regression.

When fitting mixed-effects models, it is important to carefully consider the treatment of each Level 1 variable (i.e., fixed or random slope). Treatment of each Level 1 variables was resolved empirically using planned likelihood ratios (LR) tests to determine the most parsimonious model. Results from LR tests, however, must be weighed against model complexity, as allowing too much variability at higher levels can become computationally infeasible (Bell et al., 2019). Thus, Level 1 variables were set to random only if doing so improved model fit, while also weighing the computational ramifications of doing so (see Supplementary Material). All mixed-effects models were fit using the mixed (with maximum likelihood) and melogit commands in STATA 15 (Stata, 2017). Significance tests were two-sided.

**Main effects**. Although prior work has probed the impact of individual stimulus properties on reasoning performance and, putatively, mental model formation, the present study is the first to examine the effects of a host of stimulus properties in relation to—and controlling for—each other. Thus, we first investigated the main effect of each stimulus property manipulated within the context of this study by running mixed-effects models taking the following Level 1 predictors: Number of Premises (2 or 3), Number of Dimensions (1 or 2), Relation Type (Spatial or Non-spatial), Solution (True, False, or Indeterminate), Premise Order (Continuous or Discontinuous), and Conclusion Phrasing (“A first” or “A second”; see Figure 1). Because each stimulus property was manipulated independent of all others, including all of the stimulus properties within a single model allowed us to examine the effect of each (controlling for all others) without concerns of collinearity between the different predictors.

**Interaction effects**. An extensive body of prior work has investigated the minimum number of Level 1 and Level 2 data points required to ensure unbiased estimates in mixed-effects modeling (Maas and Hox, 2005; Leon and Heo, 2009; Scherbaum and Ferreter, 2009; Peugh, 2010; McNeish and Stapleton, 2016). Although a precise consensus has not been established, a recent review of the relevant literature (McNeish and Stapleton, 2016) indicated an increased likelihood of bias when models include <10 Level 1 data points, especially for mixed-effects models with a binary dependent variable (i.e., mixed-effects logistic regression). Further, even larger sample sizes are required for interactions involving binary predictors (as is the case for all interaction models in the present study; Leon and Heo, 2009). Thus, in order to reduce the number of mixed-effects models and statistical comparisons—as well as avoid potential statistical bias stemming from insufficient Level 1 data points (i.e., too few trials for a specific condition of each binary predictor; see Supplementary Material)—we focused only the interaction between Number of Premises and Number of Dimensions. This decision stemmed from the *a priori* prediction that these two stimulus properties were likely to yield the strongest main effects, given previous work indicating that increasing the number of premises and dimensions place greater burdens on working memory and mental model formation (Johnson-Laird, 1972, 1989, 2001; Klauer, 1997; Goodwin and Johnson-Laird, 2005). It is notable, however, that despite these findings, the interaction between Number of Premises and Number of Dimensions is previously untested. That is, are reasoning problems that contain additional premises and multidimensional relations even more challenging than those with only one of these stimulus properties alone?

## Results

### Descriptive Statistics

Average accuracy and reaction time (RT) for each stimulus property can be found in Table 1. The problems with the lowest accuracy (*M* = 0.59, SD = 0.32) and longest RT (*M* = 46.48 s, SD = 40.48 s) had the following stimulus properties: two dimensions, three premises, non-spatial relations, and true solutions. The problems with the highest accuracy (*M* = 0.84, SD = 0.30) and shortest RT (*M* = 23.30, SD = 19.11) had the following characteristics: one dimension, two premises, spatial relations, and true solutions. Normative accuracy and RT data are available for all MRRT problems at https://osf.io/qfvp2/.

**Table 1:** Descriptive results for each stimulus property.

	**Accuracy (#correct/total)**		**Reaction time (seconds)**	
**Variable**	**Average**	**SD**	**Average**	**SD**
**Premises**				
Two premise	0.77	0.17	27.56	15.18
Three premise	0.69	0.17	41.21	25.88
**Dimensions**				
One dimension	0.77	0.18	30.77	19.15
Two dimension	0.70	0.17	37.99	22.48
**Relation type**				
Spatial	0.73	0.18	35.09	20.78
Non-spatial	0.72	0.17	35.98	21.54
**Solution**				
True	0.74	0.16	35.80	22.46
False	0.78	0.18	29.52	18.10
Indeterminate	0.69	0.22	36.18	21.10
**Premise Order**				
Continuous	0.73	0.19	34.47	21.48
Discontinuous	0.72	0.17	35.44	19.58
**Conclusion phrasing**				
A first	0.73	0.17	35.04	20.74
A second	0.724	0.17	35.824	20.371

### Main Effects of Stimulus Properties

We ran two mixed-effects models to examine the extent to which each of the manipulated stimulus properties impacted task performance (Model 1: Accuracy, mixed-effects logistic regression; Model 2: RT, mixed-effects linear regression). Following a series of likelihood ratio tests (see Supplementary Material), both models were fit with random slopes for Premises, Dimensions, and Relation Type. Results indicate clear evidence that a number of manipulated stimulus properties significantly impacted task performance (Tables 2, 3).

**Table 2:** Mixed-effects linear regression model for reaction time (fixed effects).

**Reaction time**	**Estimate**	**Std. Err**.	**z**	***p***	**95% Conf. interval**	
**Relation type**						
Spatial	−1.90	0.93	−2.05	0.040	−3.71	−0.08
**Premises**						
Two premises	−13.44	0.94	−14.31	<0.001	−15.28	−11.60
**Dimensions**						
One dimension	−7.24	0.96	−7.51	<0.001	−9.13	−5.35
**Premise order**						
Continuous	−0.55	1.17	−0.47	0.638	−2.84	1.74
**Solution**						
False (determinate)	−1.59	0.95	−1.68	0.093	−3.45	0.27
True (determinate)	−0.59	0.93	−0.64	0.522	−2.41	1.22
**Conclusion phrasing**						
A first	−0.20	0.95	−0.21	0.833	−2.07	1.67
Intercept	46.66	1.53	30.54	<0.001	43.66	49.65

*All variables dummy coded. Relation Type: spatial vs. non-spatial; Premises: two-premise vs. three-premise; Dimensions: one-dimension relations vs. two-dimension relations; Premise Order: continuous vs. discontinuous; Solution: False vs. Indeterminate and True vs. Indeterminate; Conclusion Phrasing: A first vs. A second*.

**Table 3:** Mixed-effects logistic regression model for accuracy (fixed effects).

**Accuracy**	**Odds Ratio**	**Std. Err**.	**z**	**p**	**95% Conf. Interval**	
**Relation type**						
Spatial	1.14	0.05	2.89	0.004	1.04	1.24
**Premises**						
Two premises	1.61	0.07	11.47	<0.001	1.48	1.74
**Dimensions**						
One dimension	1.57	0.07	10.43	<0.001	1.44	1.70
**Premise order**						
Continuous	1.07	0.06	1.31	0.189	0.97	1.19
**Solution**						
False (determinate)	1.30	0.06	6.03	<0.001	1.19	1.41
True (determinate)	1.30	0.05	6.18	<0.001	1.20	1.41
**Conclusion phrasing**						
A first	0.95	0.04	−1.26	0.209	0.87	1.03
Baseline odds	1.75	0.11	8.72	<0.001	1.54	1.98

*All variables dummy coded. Relation Type: spatial vs. non-spatial; Premises: two-premise vs. three-premise; Dimensions: one-dimension relations vs. two-dimension relations; Premise Order: continuous vs. discontinuous; Solution: False vs. Indeterminate and True vs. Indeterminate; Conclusion Phrasing: A first vs. A second*.

First, we observed a strong effect of number of premises for both accuracy and RT. Specifically, two-premise problems were associated with faster responding (estimated effect = −13.48 s, *z* = −14.31, *p* < 0.001), and participants were 1.61 times more likely to provide the correct response (relative to accuracy for three-premise problems; *z* = 11.47, *p* < 0.001). We additionally observed a strong effect for number of dimensions: participants were both faster (estimated effect = −7.24 s, *z* = −7.51, *p* < 0.001) and more accurate (OR = 1.57, *z* = 10.43, *p* < 0.001) on one-dimension reasoning problems compared to performance on two-dimension reasoning problems. Results further indicated a significant effect of relation type on RT and accuracy, such that performance was greater for problems with spatial relations (RT: estimated effect −1.90 s, *z* = −2.05, *p* = 0.04; Accuracy: OR = 1.14, *z* = 2.89, *p* = 0.004).

Given prior findings indicating that reasoning problems with an indeterminate solution are more challenging than those with a determinate solution (Byrne and Johnson-Laird, 1989; Schaeken et al., 2007), mixed-effects models also assessed the effect of solution type (with indeterminate problems entered as the reference). Results were largely consistent with previous work; participants were 1.3 times more likely to provide a correct response on True determinate problems (*z* = 6.18, *p* < 0.001) and False determinate problems (*z* = 6.03, *p* < 0.001) relative to problems with an indeterminate solution. For RT, no differences were observed for False determinate problems relative to indeterminate problems (estimated effect = −1.59 s, *z* = −1.68, *p* = 0.09) or True problems relative to indeterminate problems (estimated effect = −0.59 s, *z* = −0.64, *p* = 0.52).

Lastly, we systematically varied the order of both the premises (Continuous or Discontinuous) and Conclusion (A first, A last; see Figure 1) to account for any differences due to sequencing of information throughout the reasoning problems. Results, however, failed to identify any differences in responding based on these criteria for RT (both *p* > 0.63) or accuracy (both *p* > 0.18), suggesting that these factors are not critical to the formation of internal mental models.

### Interaction Effects of Number of Premises and Number of Dimensions

Having identified significant main effects of Number of Premises and Number of Dimensions, we next investigated interactions between these two stimulus properties. That is, were their effects on task performance even more (or less) pronounced when paired with each other? Notably, we failed to identify significant interaction effects on RT (Supplementary Material; see Discussion for consideration of RT as a performance metric in the present task).

**Figure 2:**
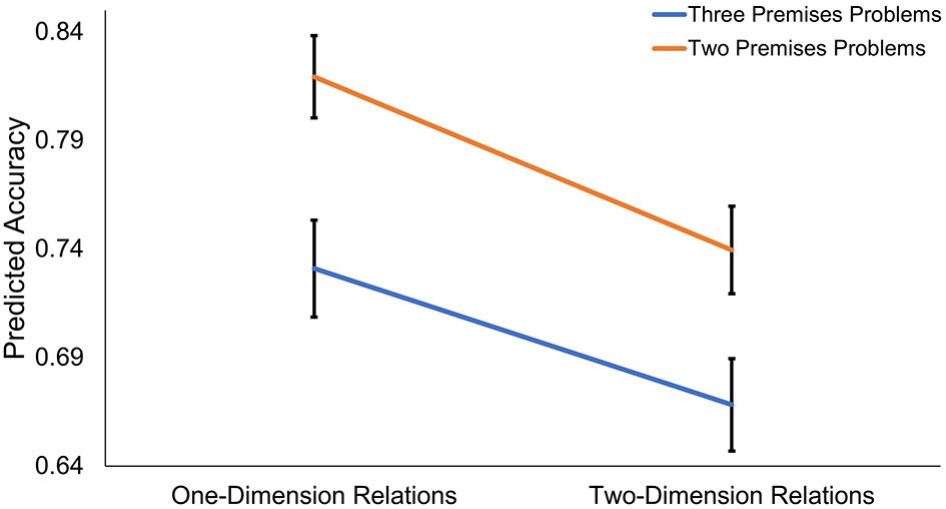
Predicted marginal means from Dimension X Premise interaction model. Results from mixed effects model indicate significant Dimension X Premise interaction. Three-premise problems with two-dimension relations associated with lowest accuracy on relational reasoning task.

Results indicated that there was indeed a multiplicative effect for accuracy (Figure 2, see Supplementary Material for more detailed model information); this model revealed a significant Number of Premise X Number of Dimension interaction (OR = 1.22, *z* = 2.64, *p* = 0.008), such that three-premise problems were associated with even lower accuracy for trials with two-dimension relations relative to the effect of three-premise problems on trials with one-dimension relations (as well as the reverse; two-dimension trials were associated with significantly lower accuracy for three-premise trials than when included in two-premise trials). That is, the most challenging problems were those that contained three premises with two-dimensional relations (e.g., “Henry is more organized and less helpful than Brian”). Two-dimensional problems are likely to necessitate the creation of more complex mental models, and the addition of a third premise (relative to two premise problems) may increase working memory load. This result indicates that performance is especially hampered when these two properties are paired together.

## Discussion

The present study investigated the extent to which a variety of syllogistic reasoning stimulus properties impact problem difficulty—putatively by interfering with the formation of internal mental models—by estimating the effects of each property through the use of mixed-effects modeling. We found that the number of premises and the number of dimensions had large effects on both accuracy and reaction time; three premise problems were more difficult than two premise problems, and problems containing two-dimensional relations were more challenging than those with relations containing only one dimension. Results further indicated that the use of spatial vs. non-spatial relations in the syllogistic reasoning problems also had a significant effect on both accuracy and reaction time; problems with non-spatial relations were more difficult than problems with spatial relations. In addition, we found that problems with an indeterminate solution were associated with lower accuracy (relative to problems with determinate solutions). There was no effect of premise order or conclusion phrasing.

Interaction analyses revealed that the most challenging problems were those that contained three premises with two-dimensional relations (e.g., “Henry is more organized and less helpful than Brian”). Number of premises and dimensions both plausibly increase task difficulty by necessitating the formation of more complex models (because more complex relations must be represented). The addition of a third premise (relative to two premise problems) is also likely to increase working memory load. It is therefore not surprising that performance is especially hampered when these two properties are paired together (i.e., there is a Number of Premise X Number of Dimension interaction).

These findings align with prior literature on relational reasoning, replicating significant effects of the number of premises (Vandierendonck, 1996; Goodwin and Johnson-Laird, 2005), the number of dimensions (Johnson-Laird, 1972, 1989) and indeterminacy (Byrne and Johnson-Laird, 1989; Schaeken et al., 2007) on problem difficulty. Prior studies have found null effects of spatial vs. non-spatial relations (Carreiras and Santamaria, 1997) however the present study is the first to examine the effects of multiple stimulus properties in relation to—and controlling for—each other within the same task. This distinction may explain why this study is the first to find significant main effects of relation type on problem difficulty.

Prior work has relied on both accuracy and reaction time to assess task performance (Johnson-Laird, 2010), thus it is worth noting that we observed stronger effects of the stimulus properties on task accuracy relative to the effects observed for reaction time. Notably, however, participants were provided with unlimited time to provide a response. We elected not to impose time constraints because stimulus properties varied widely and normative reaction times for these problems was not previously known—one goal of this study was to assess the time it took to solve each problem without constraint. Therefore, since participants had unlimited time to solve each trial, they may have spent more effort (reflected by longer response times) solving the problems that appeared more challenging upon initial presentation (e.g., because there were multiple premises and/or dimensions). Without time constraints, participants could wait to provide a response until they felt certain of their answer. It is plausible that, in a fixed-time design, greater effects on RT would emerge. Future work should examine whether the presently observed effects are also observed in a time-fixed design, and perhaps include an even greater number of problems for particular stimulus properties to enable greater consideration of potential interactions (e.g., whether the structure of the mental model in two dimensional problems impacts difficulty).

Finally, we present the MRRT, along with normed accuracy and RT data for every problem, in the hope that it may be useful for future investigations of relational reasoning (https://osf.io/qfvp2/). In particular, research aimed at testing and/or training mental model-based reasoning may benefit from the ability to manipulate difficulty along multiple stimulus property dimensions.

## Data Availability Statement

The datasets presented in this study, along with normative data for the MRRT, can be found in the Open Science Framework: https://osf.io/qfvp2/.

## Ethics Statement

The studies involving human participants were reviewed and approved by Institutional Review Board of Georgetown University. The patients/participants provided their written informed consent to participate in this study.

## Author Contributions

AEG, RAC, and ABW: conceptualization. HOK, DLW, GAC, RAC, and ABW: methodology. RAC and ABW: formal analysis. RAC and GAC: investigation. GFP: data curation. RAC, GAC, and ABW: writing—original draft preparation. AEG, RAC, ABW, HOK, DLW, GAC, GFP, and ELD: writing—review and editing. RAC, ABW, and ELD: visualization. AEG: supervision. RAC and GAC: project administration. AEG and RAC: funding acquisition. All authors have read and agreed to the published version of the manuscript.

## Conflict of Interest

The authors declare that the research was conducted in the absence of any commercial or financial relationships that could be construed as a potential conflict of interest.
