# Supplementary material for: What Makes Mental Modeling Difficult? Normative Data for the Multidimensional Relational Reasoning Task
Source: Front Psychol. 2021 May 6;12:668256. doi: 10.3389/fpsyg.2021.668256 (PMC8134533; doi:10.3389/fpsyg.2021.668256)
Supplement: Supplementary file 1 [file Data_Sheet_1.PDF]

Table S1. Average number of trials per condition for each participant

	Two Dimensions (N=42.02)		One Dimension (N=22.67)		
	Nonspatial	Spatial	Nonspatial	Spatial	
**Three Premises (N=32.68)**					*Total*
Solution: Indeterminate	3.01	4.00	1.34	2.33	*10.67*
Solution: False	3.34	4.01	2.00	1.33	*10.69*
Solution: True	3.33	3.66	1.67	2.67	*11.32*
Total	*9.68*	*11.67*	*5.01*	*6.33*	
**Two Premises (N=32)**					*Total*
Solution: Indeterminate	3.33	3.34	2.00	1.66	*10.33*
Solution: False	3.66	3.33	2.67	1.67	*11.33*
Solution: True	4.01	3.00	1.33	2.00	*10.34*
Total	*11.00*	*9.67*	*6.00*	*5.33*	

Table S2. Results of likelihood ratio tests for effects of random slopes for each Level 1 variable

	None	Random Premises	Random Dimensions	Random Relation Type	Random Solution	Random Premises & Dimensions	Random Premises & Relation Type	Random Dimensions & Relation Type
Random Premises	30.05***<br>9.34**	---	---	---	---	---	---	---
Random Dimensions	22.88***<br>6.01*	---	---	---	---	---	---	---
Random Relation Type	18.13***<br>26.23***	---	---	---	---	---	---	---
Random Solution	0<br>197.07***	---	---	---	---	---	---	---
Random Premises & Dimensions	51.571***<br>15.33***	27.67***<br>5.99*	34.84***<br>9.32**	---	---	---	---	---
Random Premises & Relation Type	49.30***<br>36.46***	19.26***<br>27.13***	---	31.18***<br>10.23**	---	---	---	---
Random Dimensions & Relation Type	45.41***<br>32.28***	---	22.53***<br>26.27***	27.29***<br>6.05*	---	---	---	---
Random Dimensions & Premises & Relation Type	81.86***<br>42.46***	---	---	---	---	24.15***<br>27.13***	32.56***<br>5.99*	36.45***<br>10.17**

Note: Pairwise results of χ^2 (from likelihood ratio test). Significant values (*** $p < .001$, ** $p < .01$, * $p < .05$) indicates fit favors random slopes for variables in left column. Variable set to random slopes only if LR test indicated significant improvement in model fit for both RT (top value) and Accuracy (bottom value).

Table S3. Random effects parameters – mixed effects linear regression for reaction time

Random Effects	Estimate	Std. Err.	95% Conf. Interval	
Premises	99.27	22.05	64.24	153.42
Dimensions	99.13	22.87	63.07	155.82
Relation Type	71.77	18.56	43.23	119.14
Intercept	443.72	44.80	364.06	540.81
Residuals	2781.74	29.04	2725.39	2839.24

Table S4. Random effects parameters – mixed effects logistic regression for accuracy

Random Effects	Odds Ratio	Std. Err.	95% Conf. Interval	
Premises	0.10	0.04	0.05	0.22
Dimensions	0.09	0.04	0.03	0.23
Relation Type	0.16	0.04	0.10	0.26
Intercept	0.70	0.07	0.57	0.86

Table S5. Mixed effects linear regression model for reaction time; Premise X Dimension interaction (fixed effects)

Reaction Time	Estimate	Std. Err.	z	p	95% Conf. Interval	
Premises						
Two Premises†	-12.63	1.09	-11.60	0.000	-14.77	-10.50
Dimensions						
One Dimension✤	-6.10	1.24	-4.91	0.000	-8.53	-3.66
Premises X Dimensions						
Two Premises: One Dimension	-2.30	1.57	-1.47	0.142	-5.37	0.77
Relation Type						
Spatial	-1.90	0.93	-2.05	0.040	-3.71	-0.09
Solution						
False (determinate)	-1.54	0.95	-1.62	0.105	-3.40	0.32
True (determinate)	-0.63	0.93	-0.69	0.493	-2.45	1.18
Premise Order						
Continuous	-0.52	1.17	-0.44	0.657	-2.81	1.78
Conclusion Phrasing						
A First	-0.29	0.96	-0.30	0.762	-2.16	1.59
Intercept	46.29	1.55	29.91	0.000	43.26	49.32

Note: † effect for two dimension relations; ✤effect for three-premise problems

Table S6. Mixed effects logistic regression model for accuracy; Premise X Dimension interaction (fixed effects)

Reaction Time	Odds Ratio	Std. Err.	z	p	95% Conf. Interval	
Premises						
Two Premises†	1.52	0.07	8.76	0.000	1.38	1.66
Dimensions						
One Dimension✤	1.44	0.08	6.68	0.000	1.29	1.60
Premises X Dimensions						
Two Premises: One Dimension	1.22	0.09	2.64	0.008	1.05	1.41
Relation Type						
Spatial	1.14	0.05	2.90	0.004	1.04	1.24
Solution						
False (determinate)	1.29	0.06	5.94	0.000	1.19	1.41
True (determinate)	1.30	0.06	6.25	0.000	1.20	1.41
Premise Order						
Continuous	1.07	0.06	1.26	0.207	0.96	1.19
Conclusion Phrasing						
A First	0.95	0.04	-1.08	0.278	0.88	1.04
Baseline Odds	1.79	0.12	9.02	0.000	1.58	2.03

Note: † effect for two dimension relations; ✤effect for three-premise problems
